# Cross-national survey data on student attitudes toward artificial intelligence

**DOI:** 10.1016/j.dib.2025.112022

**Published:** 2025-09-04

**Authors:** Ján Skalka, Małgorzata Przybyła-Kasperek, Eugenia Smyrnova-Trybulska, Cyril Klimeš, Radim Farana, Valentina Dagienė, Vladimiras Dolgopolovas

**Affiliations:** a Faculty of Natural Sciences and Informatics, Constantine the Philosopher University in Nitra, 949 01 Nitra, Slovakia; b Faculty of Science and Technology, University of Silesia in Katowice, 41-200 Katowice, Poland; c Institute of Pedagogy, Faculty of Arts and Educational Sciences, University of Silesia in Katowice, 40-007 Katowice, Poland; d Department of Informatics, Faculty of Business and Economics, Mendel University in Brno, 613 00 Brno, Czech Republic; e Institute of Educational Sciences, Vilnius University, Universiteto str. 9, Vilnius, LT-01513 Lithuania; f Institute of Data Science and Digital Technologies, Vilnius University, Akademijos str. 4, Vilnius LT-08412, Lithuania

**Keywords:** AI literacy, Student perception, Higher education, Digital skills, AI anxiety, Technology acceptance

## Abstract

This data article presents responses from a comprehensive, multi-year survey conducted between 2022 and 2024 at several universities in Central and Eastern Europe, focusing on students' artificial intelligence (AI) literacy, attitudes, and readiness. The data collection was part of a longitudinal project within the FITPED consortium, which built upon previous EU-funded initiatives to support digital education. A total of 1146 university students participated, representing a diverse range of study programs, academic years, and countries, including Slovakia, Poland, the Czech Republic, Lithuania, Indonesia, Turkey, France, and Ukraine. The structured questionnaire was based on validated instruments and included constructs such as AI literacy, AI readiness, AI anxiety, behavioural intention, satisfaction, confidence, perceived relevance of AI, and social goods. Items were rated on a 5-point Likert scale, and demographic information, including gender, age, year of study, field of study, and previous experience with AI-related courses, was collected. The survey was administered anonymously via Google Forms and the Moodle LMS in multiple languages, ensuring accessibility across disciplines. The dataset supports cross-disciplines and longitudinal comparisons and is suitable for quantitative analytical methods such as factor analysis and structural equation modeling. This openly shared dataset provides a foundation for tracking trends in AI readiness and perceptions in higher education. It allows for its reuse for comparative international research, curriculum development, and targeted educational interventions that promote inclusive and context-aware AI literacy.

Specifications Table

**Table:** 

Subject	Social Sciences
Specific subject area	Perceptions of artificial intelligence and student readiness for its implementation in higher education across disciplines and countries.
Type of data	Table (.csv format) Supporting materials (PDF version of survey)
Data collection	Data were collected through an online questionnaire, delivered via Google Forms and Moodle, from 2022 to 2024. The instrument was adapted from a validated questionnaire by Dai et al. (2020) and included items assessing AI literacy, readiness, satisfaction, relevance, anxiety, and other related constructs, all rated on a 5-point Likert scale. Participation was voluntary, and responses were anonymous for Google Forms and anonymized for Moodle LMS. Invalid and duplicate records were removed. Item-level Not applicable (N/A) responses were retained and are coded as 0 in the released file. The dataset also includes responses from 59 respondents who participated across multiple years and are linkable across waves.
Data source location	Collected: Slovakia, Poland, the Czech Republic, Lithuania, Indonesia, Turkey, France, and Ukraine. Stored: Figshare, Constantine the Philosopher University in Nitra, Slovakia
Data accessibility	Repository name: Figshare Data identification number: https://doi.org/10.6084/m9.figshare.29488523.v2 Direct URL to data: https://figshare.com/articles/dataset/AI_Literacy_Questionnaire_data/29488523/2
Related research article	none

## 1 Value of the Data

This dataset offers significant value to the scientific community and provides a starting point for various areas of research related to AI literacy and its impact on society.

• Longitudinal view of the evolution of AI perceptions – the dataset captures AI-related attitudes before and after the public surge in LLM tools, enabling comparisons across waves and within matched cohorts over a short but critical period.
• Comprehensive assessment of AI constructs – the dataset includes ten constructs related to AI perceptions, ranging from general literacy and perceived risks to societal benefits, self-rated competence, and behavioral and career intentions. This broad scope supports the exploration of complex interrelationships that go beyond fundamental descriptive analysis.
• Best use for benchmarking in similar populations – the sample is non-probability and skewed toward IT students (69.1 %) and Slovak respondents (56.9 %). It is most suitable for within-institution / IT-dominant cohort benchmarking and comparisons among similar populations; generalization beyond these groups should be cautious.
• Cross-discipline and international comparisons are feasible where group size is sufficient; we report counts by specialisation and country to define feasible contrasts. Post-stratification may only be considered if reliable external population margins are available; otherwise, reweighting is not recommended.
• Broad applicability for educational and policy research – the data can be reused by educational institutions to benchmark student readiness for AI integration, by educators to design and evaluate AI literacy curricula, and by social scientists to explore the broader societal implications of AI adoption. Policymakers can draw on the data to develop evidence-based strategies for responsible AI development and public engagement.
• Support for multivariate and model analysis – the dataset structure supports correlations, regression, EFA/CFA/SEM, and technology acceptance extensions with proper handling of N/A and reporting of effective n. It is necessary to consider the reduced n for IM, C, S, BI (due to N/A) and the marginal internal consistency of AI Literacy (L; α=0.688; and 0.743 excluding L4); model-based analyses should take these limitations into account (e.g., FIML/MI, sensitivity checks).

## 2 Background

Artificial intelligence is rapidly changing industries, the job market, and the future of professions. Universities must respond to this challenge, whether for reasons of social responsibility or to maintain their sense of existence [1,2]. The original motivation for collecting data on students' attitudes toward AI arose before the widespread adoption of LLMs. It was based on the need to understand how formal and informal education about AI influences students' attitudes and understanding of AI at different stages of their studies and completion of AI courses.

Despite the existence of several AI literacy frameworks ([[3], [4], [5]]), none of them has been directly used as the basis for designing the questionnaire. To assess students' attitudes, a validated questionnaire [6] was chosen, explicitly developed for the educational environment and based on a synthesis of educational and psychological models.

Although the questionnaire was initially designed for computer science students, its use has expanded to all study programs, in line with the growing importance of AI literacy and the provision of courses for non-computer science students. It has gradually become meaningful to collect and compare data across disciplines.

This data article enhances the related research publication by providing transparent and comprehensive documentation of the dataset. While a research article may focus on analytical results, a data article provides detailed information about the constructs, questionnaire items, and data collection.

## 3 Data Description

The documents linked to this data-in-brief article consist of [7]:

• Dataset file (*AI_Literacy_questionnaire_data_.csv*) - CSV file containing the cleaned and anonymized responses from 1146 university students collected between 2022 and 2024. Fifty-nine students completed the questionnaire twice, in 2022 and 2024. They can be distinguished or identified based on the ID in the first column of the dataset. Each row corresponds to one answer respondent, and each column represents either a demographic variable or a questionnaire item. The dataset includes:
  o User_id – a unique numerical code assigned to each respondent. This ID is used to link responses across multiple time points (e.g., for students who completed the questionnaire in both 2022 and 2024). It ensures data continuity while maintaining anonymity. Each identifier corresponds to a unique participant; unless the second response could not be reliably matched, the record was excluded.
  o Demographics: Grade (year of study), Age (in years), Gender, Country, Study programme, Number of hours of AI-related courses
    Likert-scale item responses (values 1–5) for 10 constructs: AI Literacy (L1–L5), AI Readiness (RE1–RE6), AI Anxiety (A1–A5), Confidence (C1–C5), Career Motivation (CM1–CM4), Behavioural Intention (BI1–BI5), Satisfaction (S1–S5), Social Goods (SG1–SG5), Intrinsic Motivation (IM1–IM4), Relevance of AI (R1–R6). For constructs (IM, C, S, BI), a ``Not applicable (N/A)'' option was available and is coded as 0.
  Invalid and duplicate records were removed. Item-level N/A (coded 0 for IM, C, S, BI) were retained by design.
• Questionnaire reference file (*AI_Literacy_survey.pdf*) – contains the complete list of all questionnaire items used in the study. Items are grouped by their corresponding psychological or attitudinal construct. The file also includes demographic questions and the source reference for the validated questionnaire [6], which can be used to verify item content, construct structure, and scale format.

## 4 Experimental Design, Materials and Methods

The dataset was collected as part of a longitudinal survey conducted within the FITPED consortium (https://fitped.eu) to map the attitudes, literacy, and readiness of university students in the field of artificial intelligence.

### 4.1 Questionnaire

Data collection was conducted using a standardized questionnaire, which served as the primary research instrument to measure respondents' attitudes in the field of AI. The questionnaire was created based on a validated instrument published in the study by Dai et al. (2020) [6], with slight modifications to accommodate the needs of multi-institutional data collection and a broader target group, including non-computer science students. The reasons for choosing an existing questionnaire were the following:

• Focus on the educational context – the questionnaire was originally developed specifically for the needs of research on students in primary schools in China, and its constructs reflect students' attitudes towards AI in educational and personal development, not only in a technical sense.
• Comprehensive coverage of psychological and educational constructs – the questionnaire encompasses multiple dimensions, allowing for a multidimensional analysis of attitudes and perceptions of AI from a broader perspective than just technical knowledge.
• Theoretical anchoring – the items are based on a synthesis of proven frameworks in the fields of technology, education, and psychology (e.g., Technology Acceptance Model, ARCS motivational model, Self-determination theory, and Theory of Planned Behavior), which increases their validity and applicability in interpreting data within a broader research framework.
• The questionnaire was freely available, and its psychometric profile (reliability, structural validity) was demonstrated in the original study, which was key to ensuring the quality and reproducibility of the research.

The questionnaire consisted of two main parts:

• Demographic part – included questions regarding gender, age, year of study, study program, country of study, and total number of hours of artificial intelligence courses completed during university studies (including elective or optional subjects).
• Main part with Likert-type items – included 50 items divided into 10 theoretically defined constructs (L, RE, A, C, CM, BI, S, SG, IM, R), each rated on a scale of 1–5 (1 = strongly disagree, 5 = strongly agree). For IM, C, S, and BI, respondents could select Not Applicable (N/A) if they had no experience with AI courses or had not formed a formulated opinion; N/A is coded as 0 in the published set and is treated as missing in all analyses. Scale scores and reliability are calculated only from non-zero responses, and we report the effective n for each subscale. The sample intentionally includes students who have not yet been in AI to improve representativeness of the population; the trade-off is smaller effective samples for IM, C, S, and BI.

The constructs included the following elements:

• AI literacy – the concept of literacy refers to the user's ability to access, analyze, and utilize information to achieve a specific purpose [8]. In this sense, AI literacy can be considered as a knowledge base that provides students with a practical understanding of the technology [9]. Item examples:
  o L1 I know that AI can be used for image recognition and search.
  o L3 I (will) use AI-assisted online translation.
• AI readiness – captures perceived readiness to interact with AI in academic and professional contexts, including knowledge, attitudes, and motivation. Item examples:
  o RE3 I like to use the advanced AI technology.
  o RE5 The new AI technology will stimulate my thinking.
• AI anxiety – assesses anticipatory emotional states (e.g., fear or discomfort) associated with the use or consequences of AI. Item examples:
  o A1 I am worried that AI will bring trouble to my future.
  o A5 I feel very pressured to hear about the advancement of AI technology.
• Confidence – measures belief in the ability to learn or apply AI, shaped by prior experiences with the technology. Item examples:
  o C1 I am confident of getting good grades in AI classes.
  o C4 I believe I can learn the basic concepts in the AI class well.
• AI relevance – reflects the perceived usefulness of AI in students' daily lives and their future careers. Item examples:
  o R1 I know that AI technology will change the world.
  o R3 I should learn the basics of AI.
• Career motivation – assesses the intention to acquire AI-related skills for career development. Item examples:
  o CM1 I think learning AI is helpful to my future.
  o CM3 Working in AI-related work is an interesting way to earn a living for me.
• Satisfaction in this context is derived from the ARCS motivational model (Attention, Relevance, Confidence, and Satisfaction) [10] and reflects how positively students evaluate their learning experience with AI. Item examples:
  o S1 Learning AI makes me feel very satisfied.
  o S3 I think learning AI is very interesting.
• Intrinsic motivation – captures an intrinsically motivated interest in learning AI, independent of external stimuli. Item examples:
  o IM1 I prefer AI topics that arouse my curiosity, even if they are difficult to understand.
  o IM2 I like the challenging AI courses so that I can learn new things.
• Behavioral intention – measures willingness or plans to use or engage with AI in the future. Item examples:
  o BI2 I want to pay active attention to the application of AI.
  o BI4 I plan to use AI tools to help me learn.
• Social goods – assesses beliefs about the potential of AI to contribute to societal benefits (e.g., health, education, environment). Item examples:
  o SG4 The combination of AI and design thinking can enhance my ability to help others.
  o SG5 It should be considered the interests of the majority when using AI.

The questionnaire was implemented in an online environment via Google Forms (for all institutions except those in Slovakia) or Moodle LMS (Slovakia), depending on the institutional context. It was available in multiple language versions (Slovak, Czech, Polish, English). Non-English versions were translated by bilingual domain researchers and independently checked by a second bilingual reviewer; item wording, order, and response options were kept identical across languages. No formal pilot was conducted. Scale-level internal consistency for our sample is reported in Table 1.

**Table 1:** Visual summary of questionnaire constructs.

Construct	Code Prefix	Number of Items	Scale	N/A share %	n (eligible)	Cronbach α
AI Literacy	L	5	1–5 Likert	0	1205	0.688
AI Readiness	RE	6	1–5 Likert	0	1205	0.825
AI Anxiety	A	5	1–5 Likert	0	1205	0.882
Confidence	C	5	1–5 Likert (0 = *N*/A)	41.33	707	0.865
Career Motivation	CM	4	1–5 Likert	0	1205	0.848
Behavioural Intention	BI	5	1–5 Likert (0 = *N*/A)	30.79	834	0.878
Satisfaction	S	5	1–5 Likert (0 = *N*/A)	48.13	625	0.881
Social Goods	SG	5	1–5 Likert	0	1205	0.736
Intrinsic Motivation	IM	4	1–5 Likert (0 = *N*/A)	47.39	634	0.832
Relevance of AI	R	6	1–5 Likert	0	1205	0.828
Total		50				

### 4.2 Participants

The dataset comprises responses from university students recruited between 2022 and 2024 by institutions participating in the FITPED consortium. Non-probability institutional convenience sampling across participating universities in multiple countries was used. Data collection took place in several waves during this period. Eligible respondents were enrolled university students invited to participate voluntarily through verbal announcements, university websites, or institutional email communications.

Participation was anonymous and voluntary. Students were informed about the anonymity of the survey. A section of the survey explained the voluntariness, anonymity, and consent by completion. Those interested in receiving the results could optionally provide their email addresses for the purpose of receiving the results. In both cases, all identifying information was either anonymized or excluded before analysis.

An additional item was included in the 2024 wave to determine whether participants had also completed the questionnaire in the previous 2022 wave. The group of respondents who participated in the questionnaire in 2023 was different, so there was no need to address respondent duplication this year. Since direct identification was not possible from the responses alone, this measure helped to avoid duplicate records from the same individual. If a respondent reported previous participation, efforts were made to match their responses across both years, either via email address (Google Forms) or LMS user ID (Moodle). If both responses were successful, they were linked under a common participant ID. If not, the second response was excluded to avoid duplication.

After removing invalid records, a total of 1146 unique students were identified, with 59 participants successfully matched across both waves (2022 and 2024). The total number of valid records in the dataset is 1205.

The demographic characteristics of the participants are summarized in Table 2.

**Table 2:** Demographic attributes of the respondents.

#	Profile	Frequency	Percentage
Year of data collection	2022 2023 2024	535 112 558	44.4 9.3 46.3
Gender	female male other	402 793 10	33.4 65.8 0.8
Grade	1 2 3 4 5 6 7	297 435 274 129 56 9 5	24.6 36.1 22.7 10.7 4.6 0.7 0.4
Age	<20 20 - 22 23 - 25 26 - 30 31 - 40 41+	122 706 249 51 42 35	10.1 58.6 20.7 4.2 3.5 2.9
Country	CZ FR ID LT PL SK TR UA	106 3 65 38 299 686 6 2	8.8 0.2 5.4 3.2 24.8 56.9 0.5 0.2
Study programme	IT IT education STEM education education language management other	833 22 14 188 28 52 68	69.1 1.8 1.2 15.6 2.3 4.3 5.6
hours of AI-related courses	0 1–5 6–10 11–20 21–50 51–100 100+	742 172 43 47 97 72 32	61.6 14.3 3.6 3.9 8 6 2.7

### 4.3 Procedure

In addition to providing basic demographic information, participants responded to items corresponding to the constructs described above using a 5-point Likert scale (ranging from 1 to 5). As specified above, the ``Not applicable (N/A)'' option was available only for four subscales (IM, C, S, BI) and is coded as 0. All other subscales used 1–5 only; 0 is not a valid value outside IM, C, S, and BI.

Data quality control included the removal of invalid records. Item-level N/A (0) responses were retained and are handled analytically as missing, with effective n per subscale reported in Table 1. In cases where participants reported completing the questionnaire in both 2022 and 2024, their responses were linked under a common identifier. All other data were anonymized and pre-processed using Python (cleaning and recoding).

### 4.4 Statistics

To examine the underlying structure and distribution of the collected data, descriptive statistical analysis was performed for all questionnaire items grouped according to their respective constructs. This initial statistical overview serves to evaluate the central tendency, variability, and distributional characteristics of participants' responses. It also helps to identify potential deviations from normality, which is crucial for selecting appropriate statistical methods in subsequent analyses. All calculations were performed in Python 3.10.10 using the Pandas 2.3.1, SciPy 1.16.1, and NumPy 1.26.4 libraries in Windows 11 and a Conda 23.3.1 environment.

Table 3 provides an overview of basic statistics for each item of the questionnaire, broken down by construct: for each item, we list n (eligible, non-zero responses), N/A count (zeros), mean, SD, skewness, and kurtosis with standard errors. As specified above, 0 denotes ``Not applicable (N/A)'' and was available only for IM, C, S, and BI; all descriptives are computed on non-zero responses to avoid distortion by inapplicable items.

**Table 3:** Descriptive statistics of questionnaire items (excluding N/*A* = 0): distribution of responses, average score, and normality among eligible respondents.

Construct	Item	Count (Valid Responses)	N/A Count	Mean (no 0)	Std Dev (no 0)	Skewness	Std. Error of Skewness	Kurtosis	Std. Error of Kurtosis
AI Literacy	L1	1205	0	4.559	0.727	−2.25	0.071	6.702	0.141
	L2	1205	0	4.529	0.718	−2.005	0.071	5.7	0.141
	L3	1205	0	3.954	1.018	−0.853	0.071	0.216	0.141
	L4	1205	0	3.23	1.361	−0.177	0.071	−1.219	0.141
	L5	1205	0	3.684	1.093	−0.526	0.071	−0.485	0.141
AI Readiness	RE1	1205	0	4.31	0.784	−1.423	0.071	2.946	0.141
	RE2	1205	0	4.31	0.76	−1.324	0.071	2.752	0.141
	RE3	1205	0	3.855	1.047	−0.816	0.071	0.144	0.141
	RE4	1205	0	3.877	0.943	−0.789	0.071	0.512	0.141
	RE5	1205	0	3.205	1.067	−0.094	0.071	−0.545	0.141
	RE6	1205	0	3.402	0.999	−0.363	0.071	−0.435	0.141
Relevance of AI	R1	1205	0	4.279	0.852	−1.404	0.071	2.32	0.141
	R2	1205	0	3.929	0.908	−0.793	0.071	0.578	0.141
	R3	1205	0	4.077	0.886	−1.068	0.071	1.293	0.141
	R4	1205	0	3.548	1.042	−0.437	0.071	−0.342	0.141
	R5	1205	0	3.347	0.997	−0.283	0.071	−0.099	0.141
	R6	1205	0	3.603	1.019	−0.604	0.071	−0.036	0.141
Social Goods	SG1	1205	0	3.924	0.99	−0.963	0.071	0.636	0.141
	SG2	1205	0	3.765	1.082	−0.765	0.071	−0.102	0.141
	SG3	1205	0	3.822	0.92	−0.691	0.071	0.418	0.141
	SG4	1205	0	3.594	1.009	−0.484	0.071	−0.105	0.141
	SG5	1205	0	3.77	0.877	−0.684	0.071	0.854	0.141
Career Motivation	CM1	1205	0	4.048	0.89	−1.071	0.071	1.41	0.141
	CM2	1205	0	3.687	1.048	−0.514	0.071	−0.331	0.141
	CM3	1205	0	3.393	1.163	−0.347	0.071	−0.703	0.141
	CM4	1205	0	3.534	1.035	−0.456	0.071	−0.248	0.141
AI anxiety	A1	1205	0	2.857	1.108	0.236	0.071	−0.765	0.141
	A2	1205	0	2.918	1.088	0.047	0.071	−0.759	0.141
	A3	1205	0	2.364	1.111	0.612	0.071	−0.319	0.141
	A4	1205	0	2.406	1.17	0.515	0.071	−0.643	0.141
	A5	1205	0	2.338	1.204	0.658	0.071	−0.497	0.141
Intrinsic Motivation	IM1	948	257	3.799	0.998	−0.712	0.08	0.016	0.159
	IM2	885	320	3.315	1.092	−0.2	0.082	−0.688	0.165
	IM3	877	328	3.552	1.053	−0.428	0.083	−0.366	0.165
	IM4	712	493	3.466	0.96	−0.338	0.092	−0.004	0.184
Satisfaction	S1	890	315	3.493	0.984	−0.502	0.082	0.124	0.164
	S2	692	513	3.549	0.944	−0.411	0.093	0.274	0.186
	S3	959	246	3.899	0.901	−1.061	0.079	1.564	0.158
	S4	689	516	3.44	0.923	−0.356	0.093	0.165	0.187
	S5	805	400	3.257	0.999	−0.246	0.086	−0.034	0.173
Confidence	C1	819	386	3.409	0.938	−0.291	0.086	−0.003	0.171
	C2	873	332	3.942	0.817	−0.928	0.083	1.675	0.166
	C3	857	348	3.386	1.002	−0.349	0.084	−0.239	0.167
	C4	894	311	3.89	0.821	−0.842	0.082	1.398	0.164
	C5	783	422	3.266	1.043	−0.229	0.088	−0.35	0.175
Behavioural Intention	BI1	881	324	3.518	1.001	−0.535	0.083	0.06	0.165
	BI2	1027	178	3.524	1.033	−0.529	0.076	−0.217	0.153
	BI3	1049	156	3.909	0.907	−1.033	0.076	1.344	0.151
	BI4	1051	154	3.914	0.948	−0.982	0.076	1.028	0.151
	BI5	1061	144	3.944	0.928	−0.916	0.075	0.837	0.15

Note: For IM, C, S, and BI, the value 0 denotes ``Not applicable (N/A)'' and is excluded from all statistics. Descriptives are computed on eligible respondents only.

Scale-level internal consistency (Cronbach's α) is summarized in Table 1; AI Literacy (L) is borderline (α = 0.688), and excluding item L4 increases α to ≈ 0.743.

### 4.5 Past research

Parts of the dataset described in this article were previously used in a study titled ``Artificial Intelligence Literacy Structure and the Factors Influencing Student Attitudes and Readiness in Central Europe Universities,'' published in the journal IEEE Access [11]. This study examines the structure of AI literacy and factors influencing attitudes, readiness, and perceived relevance of AI in higher education. Only a subset of the entire dataset was used, and the analysis was limited to selected constructs and items. The current dataset enables more comprehensive cross-sectional and longitudinal analyses, extending beyond the scope of the original study.

## Limitations

Several limitations should be considered when working with this dataset.

First, there is an imbalance in the temporal distribution of responses, with data from 2023 representing only 9.3 % of the total, which could limit the reliability of temporal comparisons.

Second, the dataset is significantly skewed towards IT students (69.1 %) and Slovak respondents (56.9 %). We recommend reporting subgroup-specific results (e.g., IT vs. non-IT) and application of post-stratification only if reliable external benchmarks are available.

Item-level completeness is lower for the four constructs where the N/A option was offered (IM, C, S, BI), which reduces the effective sample sizes (IM 47.39 %, C 41.33 %, S 48.13 %, BI 30.79 % N/A). The N/A option was treated as missing and was not imputed, so estimates for these scales are conditional on eligible respondents (i.e., those who have experience with the AI course or have formed an opinion), and precision is lower. Comparisons between constructs should account for differences, and results should be interpreted accordingly.

AI Literacy (L) shows borderline internal consistency (α = 0.688; *n* = 1205), which suggests limited homogeneity of items; item L4 has the weakest corrected correlation between item and total score, and removing L4 increases α to 0.743. Implications: Associations involving L are likely attenuated by measurement error, so effect sizes are considered lower-bound estimates. If possible, it is necessary to prefer latent variable models or include sensitivity analyses without L4. Future revisions should reformulate/replace L4 and reassess unidimensionality.

Additionally, several items exhibited significant deviations from normality. Items such as L1 and L2 (AI literacy) had strong negative skewness (skewness < –2) and high kurtosis (>5), while many others exhibited skewness beyond acceptable limits (>|1|) or platykurtic distributions (kurtosis < –1). These distributional issues may affect statistical methods that assume normality, such as parametric tests or structural equation modeling.

Overall, although the dataset is large and generally complete, these structural and distributional limitations should be considered in further analyses.

## Ethics Statement

This study involved the collection of anonymous questionnaire data from voluntary participants. Due to the anonymous nature of the responses, it was not possible to obtain formal written informed consent from each individual. However, participants were clearly informed at the beginning of the questionnaire about the purpose of the study, the voluntary nature of their participation, and the anonymization of their responses. No personally identifiable information was retained for analysis; optional emails (for result notification) were not included in the released dataset.

The study protocol was reviewed and approved by the Ethics Committee of the University of Constantine the Philosopher in Nitra (approval number UKF/225/2024/191013:002). All procedures were performed in accordance with the ethical standards outlined in the Declaration of Helsinki.

The authors confirm that they have read and complied with the ethical requirements for publication in Data in Brief.

## Credit Author Statement

**Ján Skalka:** Conceptualization, Methodology, Writing - Original Draft, Project administration, Funding acquisition. **Małgorzata Przybyła-Kasperek:** Data Curation, Methodology, Writing - Review & Editing. **Eugenia Smyrnova-Trybulska:** Data Curation, Validation. **Cyril Klimeš:** Data Curation, Validation, Writing - Review & Editing. **Radim Farana:** Data Curation, Validation. **Valentina Dagienė:** Data Curation, Methodology, Writing - Review & Editing. **Vladimiras Dolgopolovas:** Data Curation, Validation.

## Data Availability

FigshareAI Literacy Questionnaire data (Original data).

## Appendix Supplementary materials

Image, application 1
