# Supplementary material for: Cross-national survey data on student attitudes toward artificial intelligence
Source: Data Brief. 2025 Sep 4;62:112022. doi: 10.1016/j.dib.2025.112022 (PMC12545832; doi:10.1016/j.dib.2025.112022)
Supplement: Supplementary file 1 [file mmc1.pdf]

Information provided to participants prior to participating in the survey

Two data collection methods were used in our study: Google Forms and LMS Moodle. In both cases, participants were informed about the purpose and nature of the research before completing the questionnaire. The information was provided in the participants' national languages.

Participants were invited to participate in the survey either:

- Orally (e.g. during class or group meetings), or
- Through email invitations

both provided in their national language. The translated version of the standard invitation used in email:

```
Dear students,

Are you afraid of artificial intelligence or are you looking forward to when it
finally takes over the job market and takes over a lot of your work?

What do you think about the change in the job market in your country? What does it
mean for your future?

Participate in an international survey and share with us your views and opinions on
AI literacy.

The survey is carried out as part of the international FITPED AI project
(https://www.fitped.eu) under the auspices of the Department of Informatics of the
Faculty of Natural Sciences and Informatics.

Filling out the questionnaire is voluntary and you can stop it at any time. All
data will be anonymized.

Thank you for your time.
```

To ensure clarity and informed participation, a shorter version of the information was also displayed to participants in the digital environments where the questionnaire was administered:

Version for Google Forms (displayed at the beginning of the questionnaire):

```
Dear student,

we would like to ask you to complete the questionnaire. We value your time and
trust that your responses will give us feedback on your perception of artificial
intelligence (AI).

Participation in this survey is, of course, voluntary and anonymous. By completing
the questionnaire, you agree to the scientific processing of the obtained anonymous
data.

Thank you in advance for your cooperation.
```

Version for LMS Moodle (displayed before starting the questionnaire):

```
Dear student,

We would like to ask you to complete the question. We appreciate your time and
believe that your answers provide us with feedback on your perception of artificial
intelligence (AI) and their aspect. Participation in this survey is, of course,
voluntary and will be anonymised. By completing the questionnaire, you agree to the
scientific processing of the obtained anonymised data.

The questionnaire is implemented within the FITPED-AI project
(https://www.fitped.eu). We thank you in advance for your cooperation.

In the questions, select the option that best describes the answer.
```

Given that students were invited to the survey by specific individuals, either by email or verbally, we do not consider it necessary to include contact information for the responsible person directly in the questionnaire.
